# Lin11-Isl1-Mec3 Domain Proteins as Mechanotransducers in Endothelial and Vascular Smooth Muscle Cells

**DOI:** 10.3389/fphys.2021.769321

**Published:** 2021-11-19

**Authors:** Alexandra Sporkova, Subhajit Ghosh, Jaafar Al-Hasani, Markus Hecker

**Affiliations:** ^1^Department of Cardiovascular Physiology, Heidelberg University, Heidelberg, Germany; ^2^DZHK (German Centre for Cardiovascular Research) Partner Site, Heidelberg/Mannheim, Germany

**Keywords:** lipoma preferred partner, zyxin, mechanosensing, mechanotransduction, LIM domain proteins, vascular cells, arterial remodeling

## Abstract

Arterial hypertension is the leading risk factor for cardiovascular morbidity and mortality worldwide. However, little is known about the cellular mechanisms underlying it. In small arteries and arterioles, a chronic increase in blood pressure raises wall tension and hence stretches, namely, the medial vascular smooth muscle cells (VSMC) but also endothelial cell (EC) to cell contacts. Initially compensated by an increase in vascular tone, the continuous biomechanical strain causes a prominent change in gene expression in both cell types, frequently driving an arterial inward remodeling process that ultimately results in a reduction in lumen diameter, stiffening of the vessel wall, and fixation of blood pressure, namely, diastolic blood pressure, at the elevated level. Sensing and propagation of this supraphysiological stretch into the nucleus of VSMC and EC therefore seems to be a crucial step in the initiation and advancement of hypertension-induced arterial remodeling. Focal adhesions (FA) represent an important interface between the extracellular matrix and Lin11-Isl1-Mec3 (LIM) domain-containing proteins, which can translocate from the FA into the nucleus where they affect gene expression. The varying biomechanical cues to which vascular cells are exposed can thus be rapidly and specifically propagated to the nucleus. Zyxin was the first protein described with such mechanotransducing properties. It comprises 3 C-terminal LIM domains, a leucine-rich nuclear export signal, and N-terminal features that support its association with the actin cytoskeleton. In the cytoplasm, zyxin promotes actin assembly and organization as well as cell motility. In EC, zyxin acts as a transcription factor, whereas in VSMC, it has a less direct effect on mechanosensitive gene expression. In terms of homology and structural features, lipoma preferred partner is the nearest relative of zyxin among the LIM domain proteins. It is almost exclusively expressed by smooth muscle cells in the adult, resides like zyxin at FA but seems to affect mechanosensitive gene expression indirectly, possibly *via* altering cortical actin dynamics. Here, we highlight what is currently known about the role of these LIM domain proteins in mechanosensing and transduction in vascular cells.

## Introduction

Blood vessels are constantly exposed to mechanical forces. The physical properties of the flowing blood are sensed by the endothelial cells (EC), which, under conditions of laminar flow, adapt both functionally and structurally to alterations in unidirectional shear stress. Mechanical forces propagated along the extracellular matrix (ECM) of the blood vessel wall are also perceived by the smooth muscle cells (SMC) of the medial layer of arteries and veins. This mechanosensing ability is pivotal for normal physiology and function and allows blood vessels to dynamically adjust their structure and function to biomechanical forces generated by altered hemodynamics. When arteries are chronically exposed to elevated circumferential wall tension, such as during hypertension, the quiescent contractile phenotype of the vascular SMC changes to secretory and growth promoting (Rensen et al., 2007; Saleh et al., 2016). This chronic increase in blood pressure frequently drives an initially adaptive inward remodeling process in small arteries and arterioles that eventually becomes maladaptive due to narrowing of the lumen and stiffening of the ECM. Both processes spur an increase in peripheral resistance that ultimately contributes to a fixation of diastolic blood pressure at a supraphysiological level. In addition to vascular SMC, the increased circumferential wall tension strains the lateral EC to EC contacts thus causing a change in the phenotype of these cells that seems to support the eventually maladaptive remodeling process(es) in the wall of these blood vessels.

Focal adhesions (FA) are integrin-containing, dynamic multiprotein structures that physically link the intracellular cytoskeleton, particularly cortical actin bundles, to the extracellular matrix (Geiger et al., 2009). They are thus ideally positioned to transduce extracellular biomechanical signals to the cell interior by altering the interaction of multiple signal transduction proteins with the cytoskeleton. More than 50 proteins associate with FA, and their composition varies greatly and is highly dynamic depending on the cell type and its interaction with the environment. A large and diverse group of mechanotransducing proteins that associate with FA are LIM domain (Lin-11, lsl-1 and Mec-3)-containing proteins (Sang et al., 2014; Kadrmas and Beckerle, 2004). LIM domains are double zinc finger structures that serve as interaction sites for specific signal transduction proteins. They facilitate the assembly of multiprotein complexes, for example, at the FA, that affect the cellular phenotype by controlling cellular motility, proliferation, or apoptosis through the regulation of gene expression. This plasticity is particularly important in vascular SMC, which exhibit a contractile non-migratory phenotype in the quiescent state but are highly promigratory during vasculogenesis. In response to vascular injury or in hypertension, vascular SMC become highly migratory and growth promoting, too, but under these conditions, they may contribute to – depending on the size of the arterial blood vessel – inward (small arteries and arterioles) or outward (conduit arteries) remodeling. Raised intraluminal pressure or mitogens, such as angiotensin II, stimulate vascular SMC to increase their mass of contractile proteins. Therefore, both mechanosensing and transduction by the medial SMC in arterial blood vessels are crucial to the dynamic regulation of the cellular phenotype in the face of changing hemodynamic forces.

**Figure 1:**
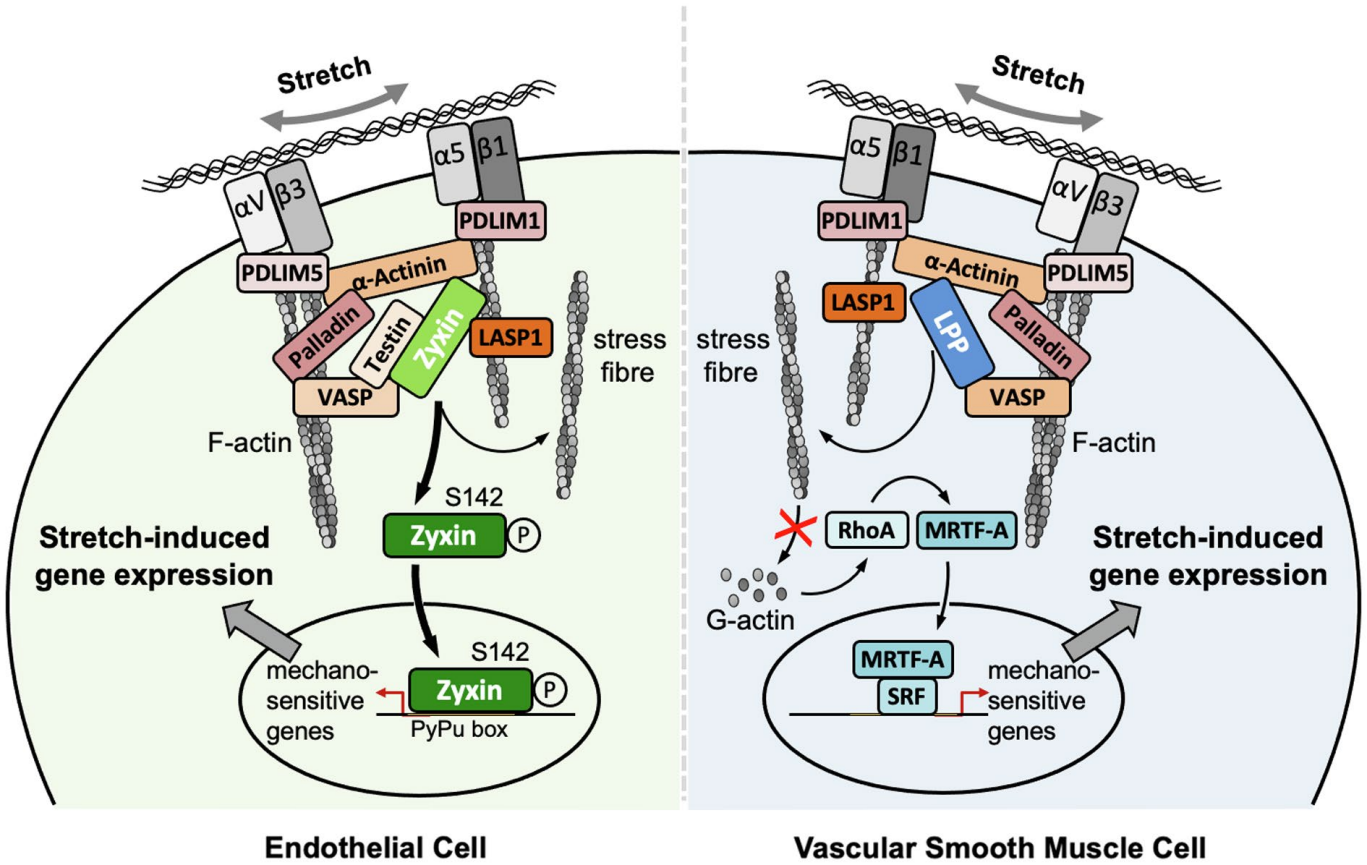
Localization and putative mechanisms of action of zyxin in EC and LPP in vascular SMC. Note that the putative mechanism of action of LPP is derived from our findings on the mechanism of action of zyxin in vascular SMC. Both LIM domain proteins seem to serve the same function in vascular SMC, that is, to maintain their quiescent contractile phenotype. Note that there is no LPP expression in adult EC of mouse or human origin. αVβ3 and α5β1, corresponding integrins; LASP1, LIM and SH3 domain protein 1; MRTF-A, myocardin-related transcription factor A; PDLIM1, PDZ and LIM domain protein 1; PDLIM5, PDZ and LIM domain protein 5; SRF, serum response factor.

Recent studies indicate that the mechanism(s) by which LIM domain proteins mediate mechanotransduction is highly conserved from yeast to mammalian cells. LIM domain-containing regions of 18 diverse LIM domain proteins have been shown to exclusively bind the stretched conformation of actin in mouse embryonal fibroblasts (Sun et al., 2020; Winkelman et al., 2020). Thus, when the actin conformation is changed by a biomechanical stimulus, specific binding sites for LIM domain proteins are unmasked. Vice versa, when actomyosin contractility is suppressed, the association of LIM domain proteins with actin and FA is also diminished (Kuo et al., 2011; Schiller et al., 2011). These findings indicate that LIM domain-mediated mechanosensing in cells is linked to the conformational state of the subset of cortical actin fibers that activate specific mechanotransduction pathways. Two structurally closely related LIM domain proteins, zyxin and lipoma preferred partner (LPP), seem to play an important functional role in the cardiovascular system. The broad spectrum of cellular pathways these proteins take part in comprises migration, proliferation, hypertrophy, and apoptosis and is a result of a large diversity of protein partners with which LPP and zyxin interact. Whereas the function of zyxin in vascular cells, especially in EC and vascular SMC, has already been well characterized (Cataruzza et al., 2004; Wójtowicz et al., 2010), the mechanotransducer role of LPP, the expression of which is restricted to SMC, in particular vascular SMC (Gorenne et al., 2003; Nelander et al., 2003), requires further investigation (*cf*. Figure 1). Interestingly, both LIM domain proteins only respond to a particular deforming stimulus, that is, increased stretch due to a rise in circumferential wall tension. Shear stress, that is, the unidirectional dragging force of the flowing blood, to which only EC are subjected, is probably too weak to elicit a translocation of zyxin from FA to the nucleus in EC, while osmotic stress does neither affect the redistribution of LPP nor that of zyxin in vascular SMC.

## Structure of Lpp and Zyxin

**Figure 2:**
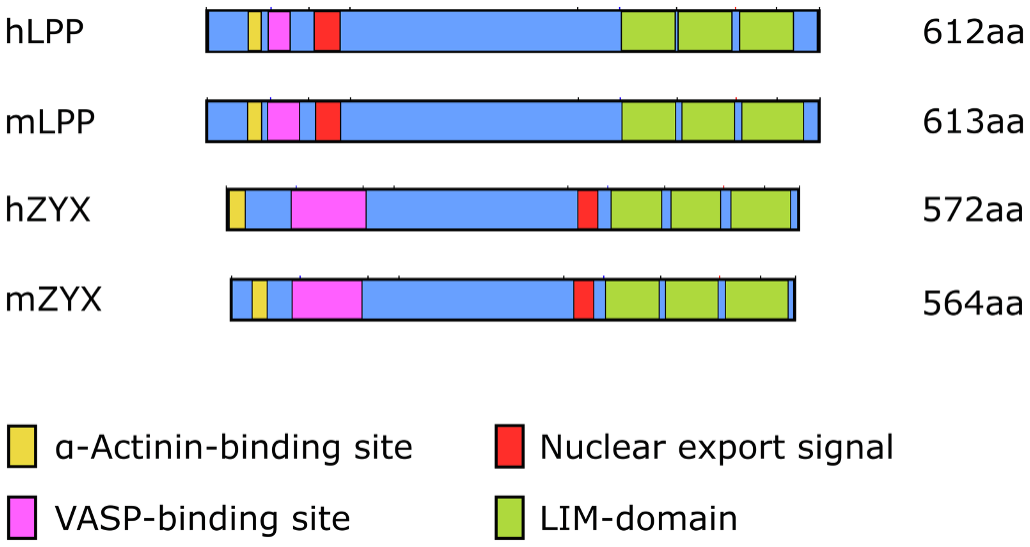
Organizational structure of human (h) and mouse (m) LPP and zyxin proteins depicting the α-actinin and VASP-binding sites close to the N-terminus as well as the three LIM domains close to the C-terminus. The length [number of amino acids (aa)] of the individual proteins is indicated on the right. Note the different localization of the nuclear export signal and the overall difference in length between LPP and zyxin in both species.

Even though LPP, on the protein level, shares only 41% sequence identity with zyxin, which in part is due to differences in length, that is, LPP is about 5% longer than zyxin both in humans and in the mouse, the organizational structure of both proteins is quite similar (Figure 2). Both zyxin and LPP contain three LIM domains at the C-terminal region and a proline-rich region (PRR) within their N-terminal sequence, which contains several phosphorylation sites and favors the formation of complexes with other proteins such as α-actinin (Petit et al., 1996). It is believed that both the LIM domain region and the PRR serve as docking sites for either rather different or the same cellular proteins (Feuerstein et al., 1994; Schmeichel and Beckerle, 1994). Moreover, zyxin contains four ActA repeats that serve as vasodilator-stimulated phosphoprotein (VASP) binding sites and one nuclear export signal (NES) between this VASP-binding region and the LIM domains (Figure 2). The nuclear export signal seems to be conserved among all zyxin family members (Siddiqui et al., 2021). There is a minor difference in the structure of the LPP protein compared to zyxin in this region in that LPP harbors only two ActA repeats, that is, VASP interaction domains, and that the single NES is located just distal to the ActA repeats rather than proximal to the first LIM domain, as in zyxin. Moreover, the PRR, which enables interaction of LPP with cytoskeletal components, including actin stress fibers and α-actinin (Li et al., 2003), lies farther from the N-terminus as in zyxin and competes for the same binding partners as zyxin but with much lower affinity.

In the cell, zyxin is mainly found at FA and along stress fibers (Nix and Beckerle, 1997). Its retention at the stress fibers is also sensitive to mechanical signals so that conditions interfering with cellular contractility result in a reduced concentration of zyxin at the stress fibers (Lele et al., 2006). In mechanically stimulated cells, zyxin directly binds to α-actinin and the cytoskeletal regulatory proteins Ena(bled)/VASP and thus contributes to the maintenance of stress fiber formation. By controlling actin assembly and function, zyxin further contributes to the structural integrity of the actin cytoskeleton (Hoffman et al., 2012). Zyxin also accumulates at sites of stress fiber damage induced by excessive strain, resulting in its rapid repair (Smith et al., 2010).

Similar to zyxin, LPP is localized to FA and cell–cell junctions, but unlike zyxin, it is only modestly expressed along stress fibers (Petit et al., 2000). At the FA, LPP binds to the ends of the actin filaments and supports cell attachment. It also interacts with VASP family members and is involved in the spatial organization of actin. In addition, the cellular distribution of LPP is affected by the biomechanical properties of the microenvironment of the cell. While LPP localizes to broad adhesion sites located toward the cell membrane when vascular SMC are grown on rigid surfaces, more elastic surfaces result in a more punctuate cellular localization of LPP (Jin et al., 2009).

**Figure 3:**
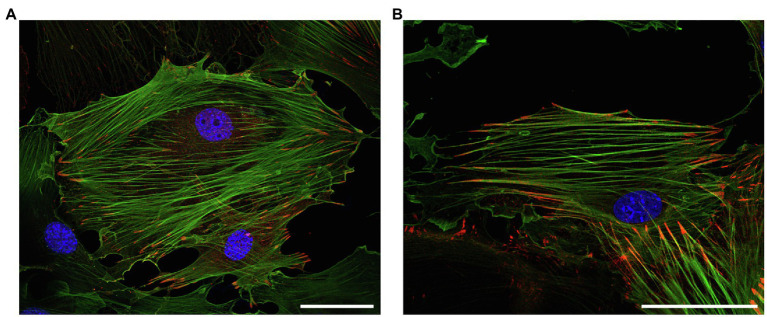
Representative immunofluorescence analysis of the intracellular localization of (top) LPP and (bottom) zyxin in cultured vascular SMC isolated from the aorta of 3-month old C57BL/6 wildtype mice. The vascular SMC (passage 3) were seeded directly on microscopic slides, fixed with *p*-formaldehyde, and stained for LPP or zyxin with a corresponding anti-LPP (HPA017342) or anti-zyxin (HPA004835) primary antibody at a dilution of 1:75 together with a primary anti-α-smooth muscle actin (α-SMA, F3777, all Sigma-Aldrich) at a dilution of 1:200. Images were recorded using a Leica TCS SP8 laser scanning confocal microscope. Both proteins (red fluorescence) mainly localize to the interface between FA and the (tips of the) cortical actin cytoskeleton, which is chiefly organized in stress fibers indicative of a mainly quiescent contractile phenotype of the cultured vascular SMC. The nuclei were counterstained with DAPI (blue fluorescence). The size marker corresponds to 50μm.

LPP and zyxin share structural characteristics (Figure 2) and binding partners (*cf*. Figure 1). They are co-expressed in vascular SMC *in vivo*, as observed in the medial SMC of larger and smaller diameter arteries in mice and humans, but not in EC (see below). Similarly, many fibroblast-like cells and epithelial cells *in vitro* co-express LPP and zyxin (Li et al., 2003). In cultured vascular SMC, LPP and zyxin are both found at the FA (Ghosh et al., 2015), more precisely at the tips of the cortical actin fibers (Figure 3), where they interact with other FA-associated proteins, such as α-actinin and VASP (*cf*. Figure 1). While this localization has been confirmed for zyxin in native murine vascular SMC (Suresh et al., 2012), this is very likely true for LPP, too, but requires experimental verification. The similar localization of both LIM domain proteins at FA in vascular SMC may have functional implications. While loss of zyxin in cultured SMC produces a shift toward the synthetic phenotype with enhanced migration of cells in a two- and three-dimensional environment as well as decreased contractility associated with improper actin assembly, overexpression of LPP in zyxin-deficient (cultured) vascular SMC fully reverts their phenotype to the quiescent contractile state (Ghosh et al., 2015). On the other hand, cultured LPP-deficient vascular SMC, which also display a synthetic phenotype, fully return to normal upon overexpression of zyxin. Due to this apparent physiological compensation, it is rather difficult to pinpoint the specific role(s) that both LIM domain proteins play in vascular SMC *in vivo*. A large network of interacting proteins could perhaps explain why zyxin cannot displace LPP from its α-actinin binding sites at FA (Li et al., 2003). This may contribute to their differential targeting to subcellular compartments and, together with the restriction of expression of LPP to (vascular) SMC in adult mice (see below), may result in significantly differing functional role(s) in the cardiovascular system.

## Expression of Zyxin and Lpp in the Cardiovascular System

**Figure 4:**
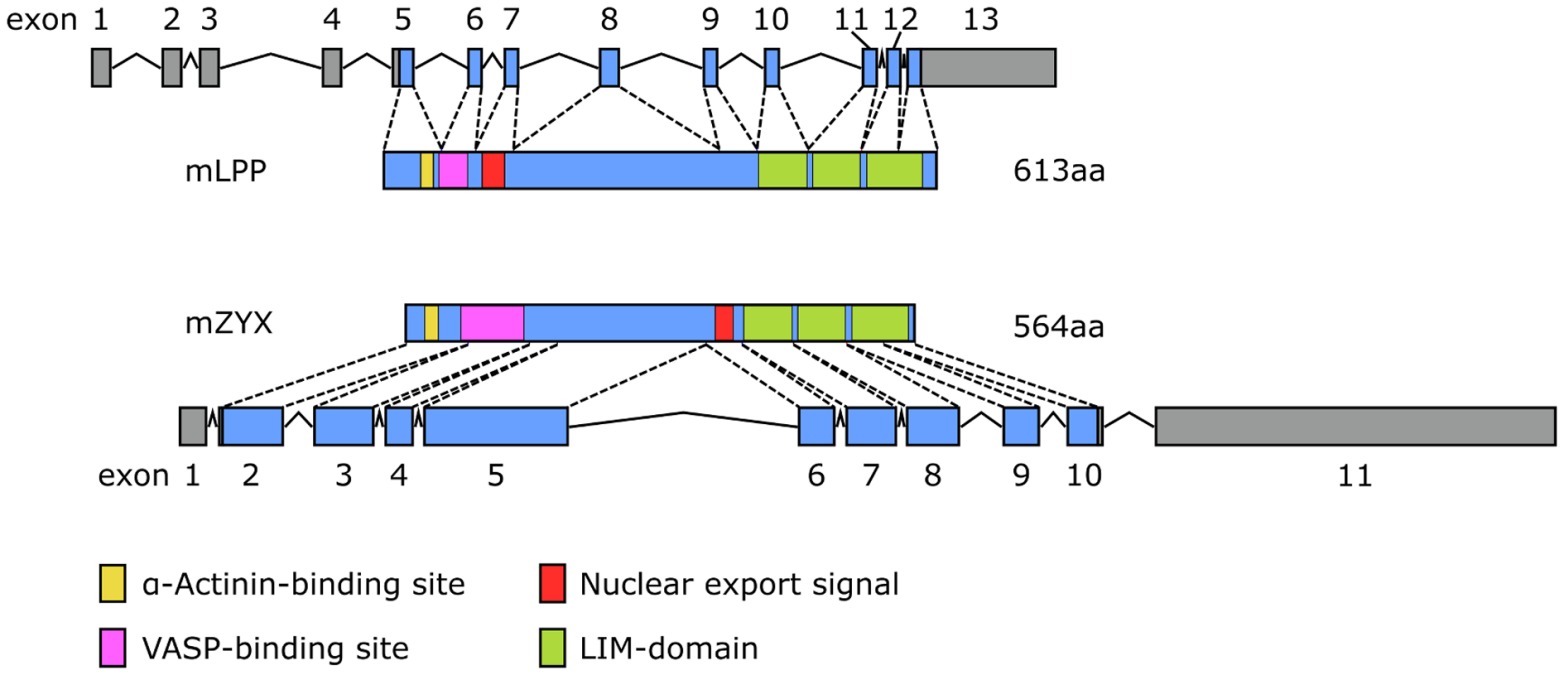
Comparison of the exon-intron structure of the primary transcripts (hnRNA) of the murine (top) *Lpp* and (bottom) *Zyx* gene and their corresponding protein products (mLPP, mZYX). The color coding for the functional elements in both proteins corresponds to that of Figure 2.

**Figure 5:**
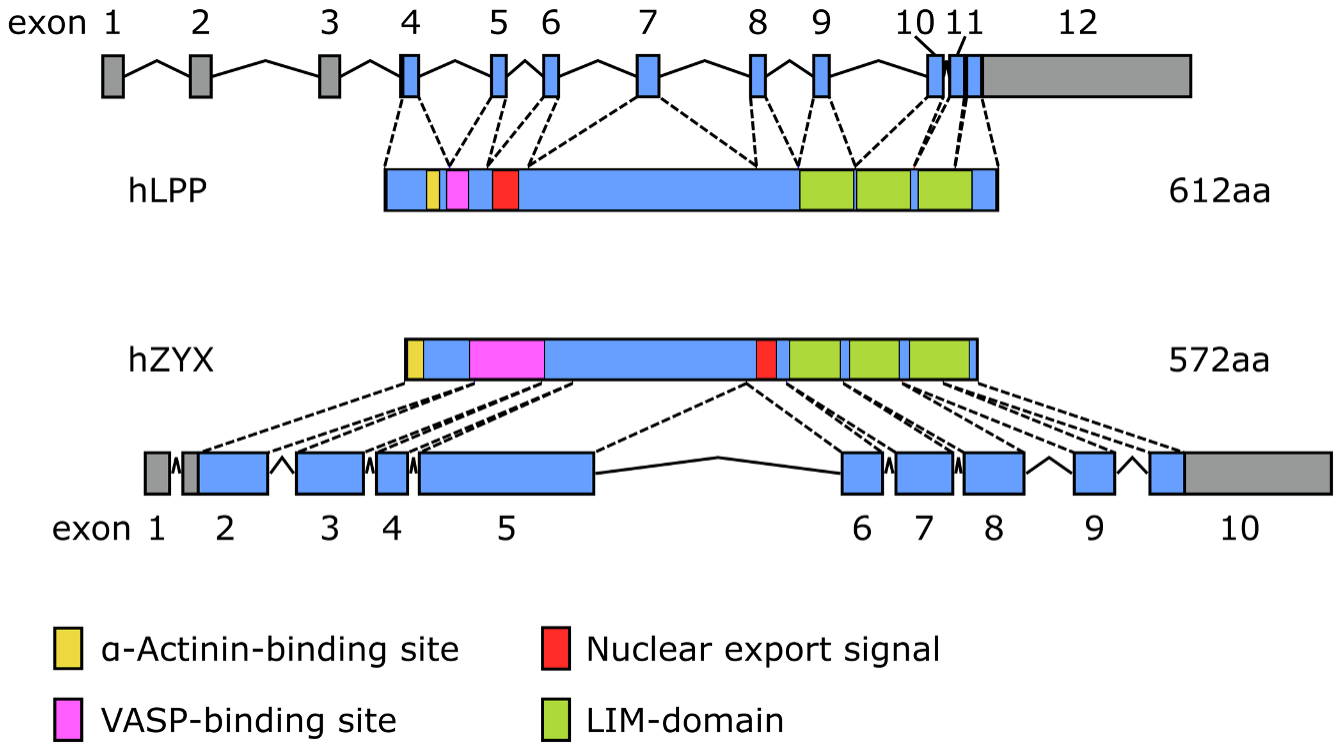
Comparison of the exon-intron structure of the primary transcripts (hnRNA) of the human (top) *LPP* and (bottom) *ZYX* gene and their corresponding protein products (hLPP, hZYX). The color coding for the functional elements in both proteins corresponds to that of Figure 2.

Besides subtle differences in their intracellular distribution, there are specific differences in the expression of LPP and zyxin that likely impact their functional role in the cardiovascular system. Studies indicate that LPP is a smooth muscle cell-specific protein in the adult tissues of several species, including mouse, rabbit, and guinea pig (Gorenne et al., 2003; Nelander et al., 2003). In the mouse, expression of the *Lpp* gene is driven by an alternative promoter in intron 2 harboring a CArG box that is an serum response factor (SRF)/myocardin-responsive element targeting *Lpp* expression to tissues with large proportions of SMC (Petit et al., 2008). As a result, translation of the protein starts rather late in exon 5 and finishes rather late near the 5′ end of exon 13 (Figure 4). The primary transcript of the human *LPP* gene looks somewhat different from the mouse hnRNA (Figure 5), there is no alternative promoter in any of the preceding introns, and translation starts in exon 4 and ends at the 3′ end of exon 11. On the other hand, translation results in a remarkably similar protein, also in terms of length.

Data from gene expression profiling studies indicate that LPP is also predominantly expressed by SMC in human tissues [these data have been deposited in the European nucleotide archive (ENA) at EMBL-EBI]. Although LPP has been detected in SMC of the urinary bladder, lung, ovaries, and gastrointestinal tract, among other tissues, a high level of LPP expression was consistently found in vascular SMC. Aorta, vena cava, saphenous vein, tibial artery, and coronary artery all show abundant expression of LPP (Bgee Database, 2021). Proteomics data show that LPP is also expressed in the atrium and the ventricles of the heart but significantly lower than in the aorta or vena cava (PRIDE database available at: https://www.ebi.ac.uk/pride/). Single-cell expression profiling data also show strong enrichment of LPP in vascular SMC as compared to, for example, cardiomyocytes (Human Protein Atlas, 2021). LPP is also expressed in several human cell types, including cardiomyocytes, EC, and fibroblasts *in vitro*, but these data have to be interpreted with caution. Cultured cells are plastic and undergo a phenotypic change associated with changes in the expression profiles of marker genes. For example, in the adult mouse, LPP is not expressed by EC (see below), whereas it is readily detected both in human and mouse EC in culture. Similarly, although LPP has been detected in cardiomyocytes *in vitro* (Hooper et al., 2012), it is not expressed by cardiomyocytes in the heart of adult mice *ex vivo* but clearly detectable in their epicardial arteries and veins (Gorenne et al., 2003). In summary, these data suggest that LPP is consistently enriched in the vasculature, in particular in medial vascular SMC in the mouse and in humans.

While zyxin appears to be expressed across different human tissues, including aorta, heart, endometrium, lung, stomach, and urinary bladder (Bgee Database, 2021), single-cell expression data consistently show enhanced expression of zyxin by the EC. This has been shown for EC of the lung, heart, eye, and liver. Similarly, zyxin is consistently expressed at high levels across several human EC types and lines. While enrichment in EC seems to be specific for zyxin but not LPP, similarly to LPP, zyxin is also expressed by vascular SMC (*cf*. Human Protein Atlas, 2021). Translation of zyxin mRNA in the mouse starts near the 5′ end of exon 2 and results in a protein that is 8% shorter than its LPP counterpart (Figure 4). Moreover, there is no alternative promoter in the preceding intron, which may explain the lack of restriction to vascular SMC in the adult mouse. The human primary transcript of the *ZYX* gene looks quite similar to its mouse equivalent and results in a protein that is slightly longer than the murine protein with the α-actinin-binding region moved right next to the N-terminus (Figure 5). It is unlikely that these subtle differences have a major impact on the tissue distribution of zyxin in mice and humans, let alone the relative enrichment in EC.

**Figure 6:**
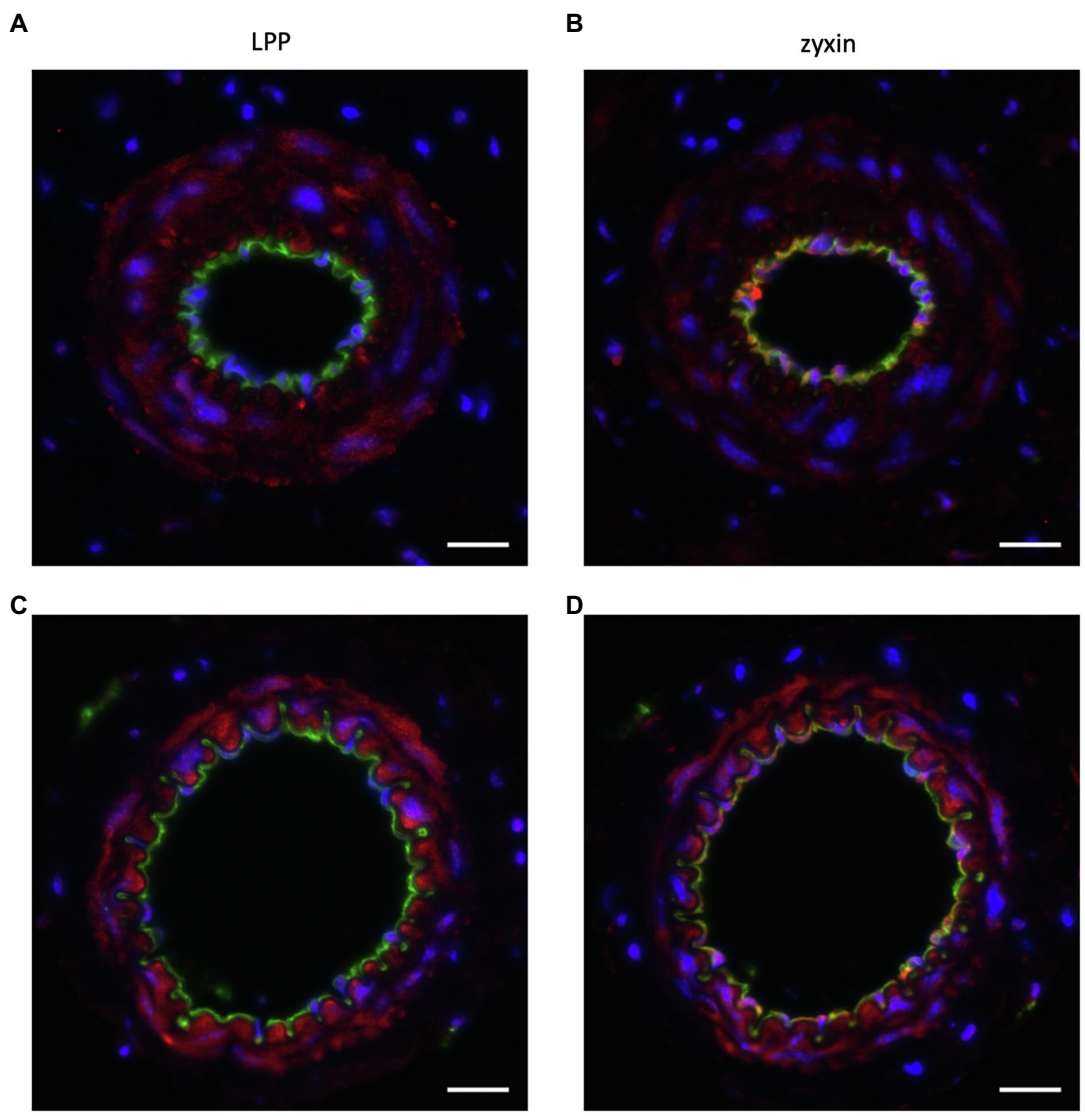
Representative immunofluorescence analysis of the cellular distribution of **(A**,**C)** LPP and **(B**,**D)** zyxin in cross sections of the **(A**,**B)** mouse femoral and **(C**,**D)** mouse mesenteric artery. The isolated blood vessels were fixed with *p*-formaldehyde overnight, embedded in paraffin, and 3 µm-thin sections were stained with primary antibodies directed against LPP (HPA017342, Sigma-Aldrich, 1:75) or zyxin (HPA004835, Sigma-Aldrich, 1:75) together with primary antibodies directed against α-SMA (F3777, Sigma-Aldrich, 1–200) or CD31 (AF3628, R&D Systems, 1:200). Nuclei were counterstained with DAPI (blue fluorescence). Images were recorded using an Olympus IX3 epifluorescence microscope. The size marker corresponds to 20μm. LPP (red fluorescence) selectively localizes to the medial SMC layer of both arteries (α-SMA-positive area, not shown), whereas zyxin (red fluorescence) is detected both in the intimal layer, that is, in the EC, and in the medial layer of both arteries. In particular in the femoral artery, there is a clear co-detection of zyxin and CD31 (green fluorescence) as indicated by the yellow fluorescence. Since CD31 is present in the EC membrane, while zyxin localizes to the cytoplasm and/or the nucleus, the remainder of the EC reveal positive CD31 immunoreactivity right next to that of zyxin. This also holds true for the mesenteric artery with some yellow fluorescence detectable, too. When compared to LPP, zyxin immunofluorescence in the medial SMC layer of both arteries is clearly weaker.

Data from our group reveal that LPP expression in the adult mouse is highly enriched in the medial layer of conduit as well as resistance-sized arteries (Figures 6A,C). On the other hand, we could not detect any LPP protein in the endothelial monolayer of these different caliber arterial blood vessels (Figure 6). This contrasts with the abundance of zyxin, which we consistently detected in the EC of both small and larger caliber arterial blood vessels (Figures 6B,D). Like LPP, zyxin is detected in the medial SMC layer of these arteries but at a (much) lower level than LPP (Figure 6).

While both zyxin and LPP play an important role in regulation of the cytoskeletal organization, bioinformatic analyses of structural data suggest that all members of the zyxin family share similarities with transcriptional regulators (Siddiqui et al., 2021), indicating that these cytoskeletal regulators may perceive the mechanical stimulation at FA and relay this information to the nucleus, presumably by different means, to accordingly affect gene expression and cellular phenotype.

## Zyxin Controls Mechanosensitive Gene Expression Both Directly and Indirectly

Zyxin was the first LIM domain protein described to shuttle between FA and the nucleus (Nix and Beckerle, 1997). It can form complexes with transcription factors and inhibit the activity of genes responsible for embryonic stem cell status implying a role in the phenotype control of cells in general (Parshina et al., 2020). In cardiomyocytes *in vitro*, zyxin translocates to the nucleus upon exposure to atrial natriuretic peptide (ANP) and subsequently activates anti-apoptotic signaling cascades (Kato et al., 2005). Furthermore, multiple studies indicate that zyxin plays an important role in transducing biomechanical cues to the nucleus in vascular SMC and EC but also in fibroblasts.

In biomechanically stimulated vascular SMC, zyxin indirectly controls the expression of mechanosensitive genes including the B-type receptor for endothelin-1 (ET_B_-receptor), tenascin-C, and plasminogen activator inhibitor-1 (PA-1; Cattaruzza et al., 2004; Ghosh et al., 2015). In fact, zyxin regulates the majority of genes (about 90%) that are affected by cyclic deformation (stretch) in vascular SMC derived from the mouse aorta (Ghosh et al., 2015). Silencing of zyxin compromises the contractility of vascular SMC (Sun et al., 2012), and on a similar note, vascular SMC derived from the aorta of zyxin knockout mice exhibit a shift from the quiescent, contractile to the activated synthetic phenotype (see above). The absence of zyxin in vascular SMC results in a more growth promoting, promigratory, and less contractile phenotype and exposure to cyclic stretch further accentuates the synthetic phenotype of these cells (Ghosh et al., 2015). This switch from the quiescent contractile to the activated synthetic phenotype is associated with major changes in the control of mechanosensitive gene expression. Enhanced RhoA activity with subsequent translocation of myocardin-related transcription factor A (MRTF-A) to the nucleus, where it associates with SRF, appears to drive adaptations in mechanosensitive gene expression that are associated with the aforementioned shift in phenotype in human or mouse cultured vascular SMC devoid of zyxin (Ghosh et al., 2015). Knockdown of MRTF-A reversed the changes in mechanosensitive gene expression in zyxin-deficient vascular SMC. While MRTF-A also accumulates in the nucleus of wild-type cultured vascular SMC upon stretch, it does so to a much smaller extent than in zyxin-deficient vascular SMC.

Zyxin also controls mechanosensitive gene expression in EC where it utilizes a quite distinct molecular mechanism and very different kinetics of activation (Wójtowicz et al., 2010; Suresh et al., 2012). Although the threshold for translocation of zyxin to the nucleus is higher in EC in culture as compared to cultured vascular SMC (Ghosh et al., 2015), both in human and mouse EC zyxin rapidly (within minutes) translocates from the FA to the nucleus upon exposure to cyclic stretch. In contrast, shear stress, irrespective of its intensity, has no effect on the nuclear translocation of zyxin in both types of EC. In the nucleus, zyxin binds to a specific *cis*-regulatory element, termed PyPu (for pyrimidine-purine) box, in the promoter of most mechanosensitive EC genes, thereby affecting their expression and apparently stabilizing their phenotype (Wójtowicz et al., 2010; *cf*. Figure 1). In essence, this effect of zyxin is very similar to its activity in vascular SMC where it stabilizes their differentiated phenotype, albeit indirectly, during supraphysiological biomechanical stress by preventing actin dynamics-driven MRTF-A-mediated mechanosensitive gene expression (Ghosh et al., 2015). Conversely, lack of zyxin in both human and mouse cultured EC gives rise to a pro-inflammatory and ECM-remodeling phenotype that may even result in endothelial-to-mesenchymal transition.

**Figure 7:**
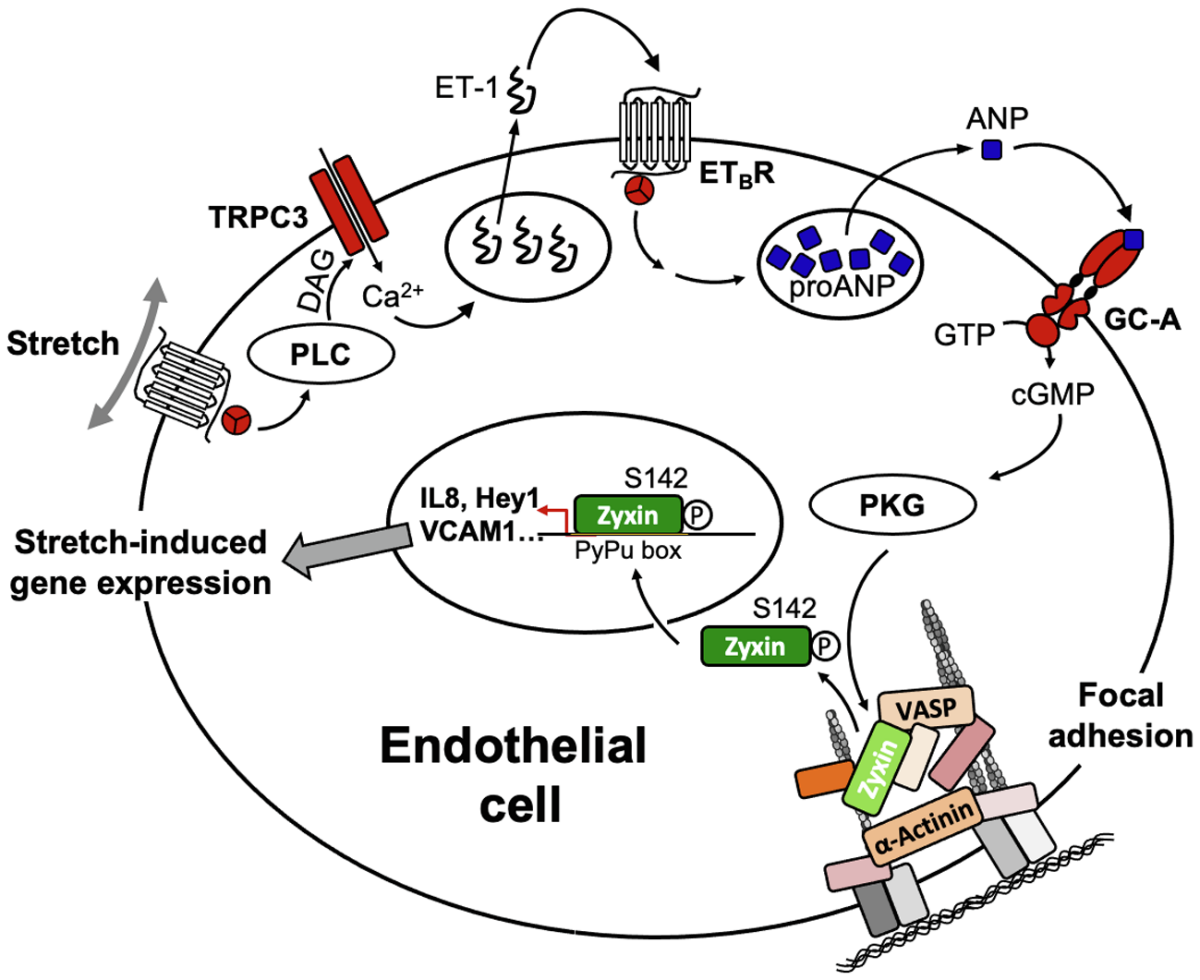
Scheme of the signal transduction cascade in EC ultimately leading to translocation of zyxin from FA to the nucleus. ANP, atrial natriuretic peptide; DAG, diacylglycerol; ET-1, endothelin-1; ET_B_R, B-type endothelin-1 receptor; GC-A, A-type guanylyl cyclase receptor; PLC, phospholipase C; PKG, protein kinase G; TRPC3, transient receptor potential cation channel subfamily C member 3; VASP, vasodilator-stimulated phosphoprotein.

The signal transduction cascade that enables zyxin to dissociate from FA and subsequently translocate to the nucleus in EC upon stretch is a rather complex multistep process. It comprises a presumably G_q_-protein-coupled receptor/diacylglycerol-mediated activation (Storch et al., 2012) of canonical transient receptor potential channel type 3 (TRPC3) triggering a small influx of extracellular calcium that leads to the release of preformed endothelin-1 from intracellular stores. Binding of endothelin-1 to the G_q_-protein-coupled ET_B_-receptor on the surface of the EC reinforces the rise in intracellular calcium, which in turn causes the release of ANP from the endothelial cells. Binding of ANP to the A-type guanylyl cyclase receptor (GC-A or NPR-A) causes activation of protein kinase G, which in turn phosphorylates zyxin at serine 142 thereby enabling it to dissociate from the FA and translocate to the nucleus of the EC (Wójtowicz et al., 2010, Suresh et al., 2012; *cf*. Figure 7).

**Figure 8:**
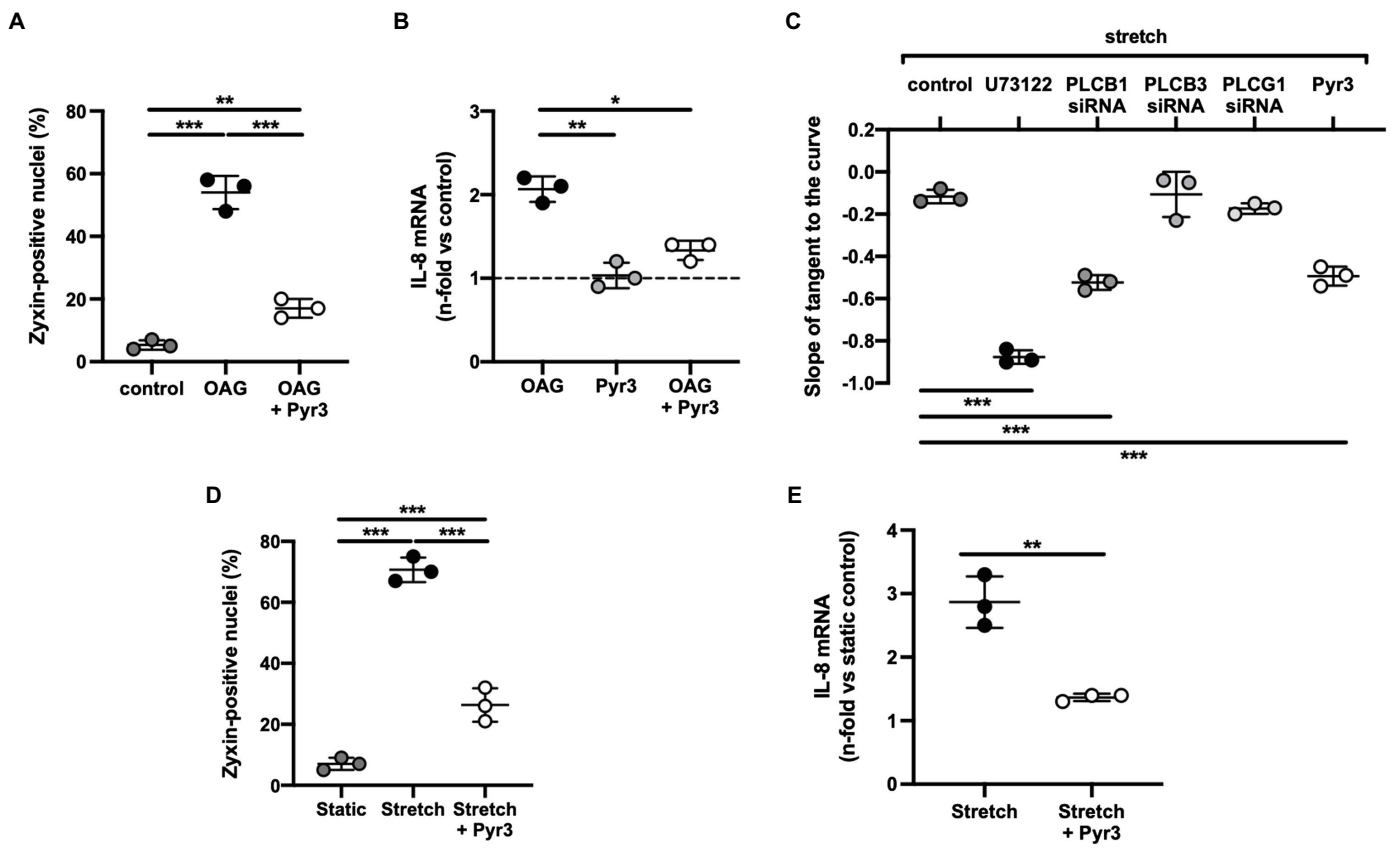
**(A)** 1-Oleoyl-2-acetyl-sn-glycerol (OAG)-induced activation of zyxin in human umbilical vein endothelial cells (HUVEC). Summary of the quantitative (immunofluorescence) analysis of zyxin-positive nuclei in control cells (passage 1) and after treatment with the diacylglycerol analogue OAG (100μm) alone for 24h or following a 6h pre-incubation with the TRPC3-selective inhibitor Pyr3 [ethyl-1-(4-(2,3,3-trichloroacrylamide)phenyl)-5-(trifluoromethyl)-1H-pyrazole-4-carboxylate; 10μm]. A primary rabbit polyclonal anti-zyxin antibody (Suresh et al., 2012) was used at a dilution of 1:250. Images were obtained using an Olympus IX81 confocal microscope equipped with an IXDSU disk unit and analyzed by using the cell^^^R software package. Two-way ANOVA with Sidak post-test for multiple comparisons, ^**^p<0.01, ^***^p<0.001 as indicated, *n*=3 for each group. **(B)** Analysis of IL-8 gene expression by real-time RT-PCR analysis (Ghosh et al., 2015) in passage 1 HUVEC following the same treatment, ^*^p<0.05, ^**^p<0.01 as indicated, *n*=3 for each group. The dashed line represents the IL-8 mRNA level in control cells under static conditions. **(C)** Cyclic stretch-induced calcium transients in Fura-2-loaded HUVEC (passage 0) stretched for 50cycles using elastic membranes. Five µM each of U73122 (pan-phospholipase C inhibitor) and Pyr3 was used. In addition, cells were transfected with siRNA against phospholipase Cβ_1_, β_3_ and γ_1_ (resulting in a 42, 35 and 38% knockdown, respectively) followed by the same stretch protocol. Quantitative analysis of the rate of decay (phase II) of the stretch-induced calcium transient by ratiometric imaging, ^***^p<0.01 as indicated, *n*=3 for each group. **(D)** TRPC3 channel inhibition by Pyr3 strongly reduces stretch-induced zyxin activation in HUVEC. Quantitative summary of the percentage of zyxin-positive nuclei in control HUVEC (passage 1) under static conditions or following 6h of cyclic stretch without or with prior treatment by Pyr3 (10μM). HUVEC were seeded onto collagen I-coated BioFlex elastomers (Flexercell) and stretched or not with a sinusoidal profile at 0.5Hz and 10% cyclic elongation. ^***^p<0.01 as indicated, *n*=3 for each group. **(E)** Analysis of IL-8 mRNA abundance in HUVEC (performed as described under B) following the same treatment. Two-tailed student’s *t*-test, ^**^p<0.01, *n*=3 for each group.

The way by which TRPC3 gets activated in human stretched cultured EC seems to comprise a G_q_-protein-coupled receptor – which is not the angiotensin II type-1 receptor due to lack of effect of losartan – that in turn causes activation of phospholipase Cβ_1_ (*cf*. Figure 8C) resulting in the formation of diacylglycerol and inositol-1,4,5-trisphosphate. TRPC3, 6, and 7 are DAG-sensitive (Dietrich et al., 2005), and among those, TRPC3 has been identified by us (Suresh et al., 2012) to be the TRP channel in cultured human and native mouse EC responsible for initiating the stretch-dependent translocation of zyxin to the nucleus. TRPC3 activation by stretch can be mimicked by using the DAG analogue 1-oleoyl-2-acetyl-sn-glycerol (OAG), and exposure of EC to OAG results in rapid translocation of zyxin to the nucleus, which is virtually abolished by the selective TRPC3 inhibitor Pyr3 (Figure 8A). Likewise, OAG-induced expression of a prototypic target gene of zyxin in EC, *CXCL8* encoding the CXC chemokine interleukin-8 (IL-8), is strongly inhibited following TRPC3 blockade with Pyr3 (Figure 8B). In addition, Pyr3 strongly inhibits nuclear translocation of zyxin as well as stimulation of *CXCL8* expression in human EC in response to cyclic stretch (Figures 8D,E), reinforcing the notion that a G_q_-protein-coupled receptor in EC acts as a mechanosensor, which *via* DAG-dependent activation of TRPC3 triggers the phosphorylation and nuclear translocation of zyxin. In the nucleus, zyxin seems to act as a transcription factor responsible for the direct transactivation (or repression) of a multitude of mechanosensitive genes that results in a stabilization of the EC phenotype when blood pressure is chronically increased (*cf*. Figure 7).

Altogether, these data indicate that zyxin is an important mechanotransducer strategically localized to the interface between the ECM and the cytoskeleton that directly influences the phenotypic properties of EC and indirectly those of vascular SMC in response to changes in their biomechanical environment, notably exposure to supraphysiological tensile stress.

## Role of Lpp As a Mechanotransducer

The role of LPP in mechanotransduction in vascular cells has not yet been described in such detail as that of zyxin. This is mainly due to the lack of the corresponding knockout mice, which ideally should be raised on the same genetic background as the zyxin knockout mice (C57BL/6), and an in-depth characterization of their respective phenotypes *in vivo*. Essentially, all knowledge about LPP to date originates from *in vitro* work with cultured cells or cell lines. However, when considering that LPP, in contrast to zyxin, is highly enriched in SMC, and in particular in vascular SMC (Gorenne et al., 2003; Nelander et al., 2003; Petit et al., 2008), in both mice and humans (see above), and its established role in the proliferation and migration of tumor cells, its role in the vasculature as a modulator of the SMC phenotype under tensile stress surely deserves proper attention.

## Control of Lpp Expression

In vascular SMC, myocardin is a potent co-activator of SRF. SRF in turn activates expression of a subset of SMC-specific marker genes and also enhances LPP expression (Gorenne et al., 2003, 2006). This activation depends on the RhoA/ROCK pathway, and accordingly, inhibitors of this pathway decrease myocardin expression and, among other SMC-specific genes, expression of the *Lpp* gene, too, suggesting that the *Lpp* gene shares transcriptional regulatory mechanisms common to SMC-specific genes. Increased activity of focal adhesion kinase (FAK) also positively regulates expression of the *Lpp* gene (Gorenne et al., 2003; Jin et al., 2007). Accordingly, FAK-deficient vascular SMC express lower levels of LPP protein and exhibit impaired migration. However, when the transcription factors Prx1a and 1b are overexpressed in these cells, abundance of LPP is increased, suggesting that *Lpp* expression is also regulated by Prx1 homebox transcription factors (Jin et al., 2007). Both Prx1a and 1b are highly expressed in the developing cardiovascular system, especially in larger arteries and veins. It is interesting to note that *Prx1* knockout mice display abnormal morphologies of the greater blood vessels (Bergwerff et al., 2000). One can only speculate at this point whether products of genes under the control of Prx1, possibly including *Lpp*, regulate proper migration of vascular SMC during vasculogenesis and that their absence thus leads to structural abnormalities of the developing blood vessels.

Although expression of LPP is very low in the adult heart as compared to that in vascular SMC (Petit et al., 1996; Human Protein Atlas, 2021), and according to our own data essentially confined to the conduit and resistance-sized arteries and veins in the adult mouse heart, pressure overload of the left ventricle caused by aortic constriction in the rat seems to increase LPP expression in the whole myocardium (Hooper et al., 2012). It should be noted, however, that in this study, LPP protein abundance in neonatal ventricles was severalfold higher than in the adult ventricles and that there was not much of a difference in LPP protein between the (unaffected) right and the (presumably hypertrophic) left ventricle in the adult rats. Since all other data were obtained with cardiac cells *in vitro*, it remains to be determined whether and in which cell type LPP provides a link between cytoskeletal remodeling and mechanical overload in the heart. In another study by the same group, knockdown of LPP by small interfering RNA impaired the adaptive response of neonatal rat cardiomyocytes to mechanical overload by compromising myofibrillogenesis (Hooper et al., 2013). Whether LPP plays a similar protective role in adult cardiomyocytes or *in vivo* is not known.

## Lpp Regulates Gene Expression and Cell Migration

One plausible mechanism how LPP may mediate cytoskeletal remodeling in response to mechanical stimuli is by activation of specific response genes that mediate these structural adaptations. LPP can shuttle to the nucleus and exhibit gene transcription activation capacity *in vitro* as measured by using a GAL-4 transactivation assay in HeLa cells. This is mediated by the two LIM domains and the proline-rich region (Petit et al., 2000). Upon exposure of cells to biomechanical deformation, LPP only transiently translocates to the nucleus where it may exhibit some transactivation capacity or function as a transcriptional co-activator to mediate the adaptive changes in gene expression in response to mechanical stress (Siddiqui et al., 2021). When the nuclear export sequence is removed or the nuclear export signal CRM1 is inhibited, LPP accumulates in the nucleus (Petit et al., 2000), emphasizing the nuclear-cytoplasmic shuttling role of LPP.

LPP is also capable of enhancing expression of PEA3-dependent genes *in vitro* (Guo et al., 2006) that are involved in neuronal development or mediation of fibroblast growth factor signaling (Roehl and Nüsslein, 2001; Firnberg and Neubüser, 2002; Livet et al., 2002). In addition, PEA3 controls genes encoding certain matrix metalloproteinases (MMP) involved in degradation of the ECM (Grunewald et al., 2009). ETV5 is a member of a PEA3 subfamily and a partner of LPP in controlling transcription. Together with LPP, it induces re-organization of cell–cell and cell-substrate contacts and promotes cell migration. In the context of cancer progression, where uncontrolled migration of and invasion by tumor cells is a pivotal feature, this effect of LPP on ETV5-dependent gene expression may reinforce epithelial-to-mesenchymal transition and expansion of the tumor into neighboring tissues (Colas et al., 2012). However, inadequate migratory behavior is associated with other pathologies as well, including atherogenesis and maladaptive hypertension-induced arterial remodeling.

Originally, LPP was discovered in a subset of lipomas as a fusion protein with high mobility group A2 (HMGA2). This fusion protein, in which the carboxy-terminal LIM domains of LPP are fused to the amino-terminal end of HMGA2, has been shown to facilitate the migration of tumor cells (Grunewald et al., 2009). In the cardiovascular system, modulating the phenotype of vascular SMC and degradation of the ECM are important steps for these cells to migrate from the media to form a sub-endothelial neointima following vascular injury (Owens, 1995). In fact, several studies have suggested a role for LPP in the migration of vascular SMC. These cells are capable of perceiving changes in stiffness and composition of the ECM. When grown on denatured collagen type 1 that simulates the microenvironment in an injured conduit artery (or vein), they upregulate expression of the *LPP* gene (Jin et al., 2009). A similar situation occurs in arterial blood vessels where both LPP and one of its preferred binding partners, paladin, are highly abundant not only in the media but also in the neointima of injured conduit arteries, again implying a functional role of LPP in the migratory behavior of vascular SMC (Gorenne et al., 2006; Jin et al., 2007). In medial SMC invading the neointima, LPP together with palladin is recruited to podosomes, that is, motile and highly dynamic actin structures found in vascular SMC in atherosclerosis (Jin et al., 2009).

In the context of tumor progression and spreading, LPP may in fact facilitate the abnormal migratory behavior of cancer cells by playing a regulatory role in the formation and function of invadopodia that promote metastasis (Ngan et al., 2017). When overexpressed in HIVS-125 cells (human SMC line), LPP enhances migration and cell spreading (Jin et al., 2007), and when LPP expression is attenuated, the invasiveness of tumor cells is also decreased (Grunewald et al., 2009). These findings suggest that the mechanosensory and perhaps mechanoregulatory function of LPP can also be employed by such cells in a pathological setting. It should be emphasized, however, that none of these findings have been obtained with cells or tissues derived from, for example, mice deficient for LPP. Moreover, *in vivo* or *ex vivo* studies of *Lpp* knockout mice are necessary to shed more light on the physiological role of LPP in blood vessels because studies with cultured cells may not adequately reflect the physiological conditions. This is especially noteworthy with regard to *Lpp* gene expression in adult mice being restricted to SMC, in particular to vascular SMC (see above). In addition, studies with cells that overexpress LPP do not accurately reflect the physiological role of LPP as overexpression of LPP disrupts normal LPP signaling and leads to a concentration-dependent upregulation of PEA3-dependent gene products including, for example, various MMP. Overexpression of these proteases would favor ECM destabilization and cell migration as frequently observed in different cancer cell lines (Guo et al., 2006; Grunewald et al., 2009). Another aspect to consider when interpreting *in vitro* findings is that vascular SMC attain a fibroblast-like phenotype in culture and that expression of characteristic marker genes and transduction pathways is modified in cultured cells (Jackson et al., 1997). This may be particularly true for LPP, which also belongs to the core of SMC-specific genes.

In contrast to its promigratory effect in LPP-overexpressing cultured cells or cancer cell lines, LPP may act as a fine-tuning protein regulating the migratory behavior of vascular SMC in response to biomechanical stimulation. While facilitating migration during vascular injury or vasculogenesis when SMC transit to a promigratory and growth-promoting state, in the absence of a specific biomechanical stimulus, LPP may support gene expression and signaling pathways associated with the quiescent contractile state. In fact, LPP was shown to act as a suppressor of cell migration associated with malignant cancer phenotypes. In lung cancer cells, MMP15 is a direct transcriptional target of LPP and ETV5. MMP15 degrades N-cadherin and weakens cell–cell contacts and thus can attenuate migration (Kuriyama et al., 2016). Similarly, recent studies suggest that a dual role of LIM proteins is plausible (promigratory vs. anti-migratory). As an example, four and a half LIM domains protein 1 (FHL-1) can act both as a tumor suppressor and a growth-promoting signaling molecule, depending on its association with diverse cellular signals (Wei and Zhang, 2020).

In fact, zyxin has also been shown to exhibit opposing actions on the migratory behavior of cells depending on the cellular context. Overexpression of zyxin in human hepatocellular cancer cells was associated with increased invasiveness and migratory abilities of these cells (Sy et al., 2006). In contrast, zyxin attenuated tumor growth, reconstituted FA organization with the actin cytoskeleton and decreased cell motility in a Ewing sarcoma model (Amsellem et al., 2005). This second action of zyxin would correspond with our results from zyxin-deficient vascular SMC that display increased migration and proliferation and poor contractile capacity, but upon re-expression of zyxin or LPP switch their phenotype back to the quiescent contractile state (Ghosh et al., 2015).

## Conclusion

In summary, LIM domain proteins, such as zyxin and LPP act, depending on the type of vascular cell, as mechanotransducers directly (zyxin) or indirectly (LPP) propagating the biomechanical stress signal into the nucleus and a change in gene expression that aims at maintaining the cells in a quiescent state. While expression of LPP seems to be restricted to vascular SMC in the adult, zyxin is highly abundant in EC. LPP presumably indirectly, *via* activation of MRTF-A relays changes in biomechanical load on the actin cytoskeleton and ECM-cell contacts (FA) to the nucleus. By acting as a sensor of the state of the cortical actin cytoskeleton, it modulates its structure and function under the complex and variable conditions of biomechanical stimulation. Markers of the differentiated state of vascular SMC are highly dependent on cytoskeletal dynamics so that, depending on the (patho)physiological context, corresponding changes in gene expression support the cells to remain in a quiescent contractile state. Zyxin, on the other hand, plays a dominant role as a mechanotransducer in EC that translocates from the FA to the nucleus where it can directly transactivate mechanosensitive genes, but has also some functional overlap with LPP in vascular SMC. Considering the presumably complex role these two mechanotransducers play in the cardiovascular system, more mechanistic studies but also animal experimental *in vivo* models are required to delineate the mechanisms through which these LIM domain proteins stabilize the quiescent phenotype of these cells in the face of a supraphysiological prolonged tensile stress as it occurs, for example, in arterial hypertension. This may eventually pave the way toward therapeutic strategies aiming at maintaining expression of these LIM domain proteins in vascular cells.

## Author Contributions

AS and MH conceived and wrote the manuscript. SG and JA-H performed experiments and contributed the data. JA-H contributed part of the figures. All authors provided critical feedback and contributed to the final manuscript.

## Funding

This work was supported by an individual research grant from the Deutsche Forschungsgemeinschaft to MH (HE 1587/12–1).

## Conflict of Interest

The authors declare that the research was conducted in the absence of any commercial or financial relationships that could be construed as a potential conflict of interest.

## Publisher’s Note

All claims expressed in this article are solely those of the authors and do not necessarily represent those of their affiliated organizations, or those of the publisher, the editors and the reviewers. Any product that may be evaluated in this article, or claim that may be made by its manufacturer, is not guaranteed or endorsed by the publisher.
